# Tuberculosis in adolescence: an integrative review focusing on patient-centered care

**DOI:** 10.1590/1984-0462/2024/42/2023027

**Published:** 2023-10-23

**Authors:** Clara Carvalho Mendes, Roberto José Gervasio Unger, Tania Cremonini de Araújo-Jorge, Anna Cristina Calçada Carvalho

**Affiliations:** a Fundação Oswaldo Cruz, Rio de Janeiro, RJ, Brazil.; b Universidade Federal do Rio de Janeiro, Rio de Janeiro, RJ, Brazil.

**Keywords:** Tuberculosis, Adolescent, Patient-centered care, Integrative review, Tuberculose, Adolescente, Assistência centrada no paciente, Revisão integrativa

## Abstract

**Objective:**

To identify how patient-centered care has been addressed in tuberculosis studies with adolescents.

**Data source:**

We searched for articles published in Portuguese, Spanish and English in the Virtual Health Library (LILACS), PubMed (MedLine), and Scopus (Elsevier) databases, from 2000 to 2020, using descriptors (DeCS, MeSH) in Portuguese and English.

**Data synthesis:**

1,322 studies were identified, of which 18 were selected. The main themes found were related to adherence to tuberculosis treatment, knowledge, attitudes and practices, health education, and public policies.

**Conclusions:**

We observed that both the number of researchers dedicated to the topic and the presence of a truly person-centered view are still scarce elements in tuberculosis among adolescents research.

## RESUMO

**Objetivo:**

Identificar, por meio de uma revisão integrativa, como o cuidado centrado no paciente tem sido abordado nos estudos de tuberculose com adolescentes.

**Fontes de dados:**

Buscamos artigos publicados em português, espanhol e inglês nas bases de dados da Biblioteca Virtual em Saúde - BVS (LILACS), PubMed (MedLine) e Scopus (Elsevier), de 2000 a 2020, utilizando descritores (DeCS, MeSH) em português e inglês.

**Síntese dos dados:**

Foram identificados 1.322 estudos, dos quais 18 foram selecionados. Os principais temas encontrados foram relacionados à adesão ao tratamento da tuberculose, conhecimentos, atitudes e práticas, educação em saúde e políticas públicas.

**Conclusões:**

Observamos que tanto o número de pesquisadores dedicados ao tema quanto a presença de uma visão verdadeiramente centrada na pessoa ainda são elementos escassos na pesquisa da tuberculose entre adolescentes.

## INTRODUCTION

Tuberculosis (TB) remains a global public health problem. Before the Covid-19 pandemic, the disease was the main cause of death by a single infectious agent.^1^

In 2014, the World Health Organization (WHO) developed a strategy named *The End TB Strategy,* ^2^ which has been successively endorsed by Brazil.^3^ It is based on three pillars, the first refers to integrated and patient-centered care and prevention. The concept of patient or person-centered care, created by the American psychologist Carl Rogers, has expanded to several areas of health and received contributions from other professionals, such as Balint, who introduced the patient-centered medicine, and Engel, who formulated the biopsychosocial model.^4,5^

Patient or person-centered care means a more holistic view of the person, considering their physical, social, cultural, and psychological aspects, their family and community needs and preferences. This approach seeks to offer empowering care, in which the subjects are active in their health and treated with respect and compassion.^2,5^ In this approach, the health professional or educator creates an environment in which three attitudes are present: empathy, acceptance of the other without judgment, and authenticity. As a result, perception increases, people learn significantly and tend to develop more and with increasing self-confidence, choosing more constructive paths and attitudes.^6,7^

Patient-centered care (PCC), according to WHO recommendations, should be used in interventions aimed at increasing adherence to TB treatment through education and communication strategies for patients and healthcare teams, offering material and psychological support for those undergoing treatment.^8^

Adolescence is defined as a process of biopsychosocial maturation that causes profound physical, social, neurological, and emotional changes in the person. Rebellion, emotional instability, increased need for acceptance and belonging to a group, search for satisfaction and differentiation in relation to parents are characteristics commonly described in this phase.^9–11^ Therefore, there is no static definition, as adolescence is a socially constructed period.

For WHO, adolescents are individuals between 10 and 19 years old. However, data on adolescents were recorded in the age groups 5–14 years and 15–24 years.^12^ It was only in 2020 that data on TB in children and adolescents were divided into age categories (0–4 years, 5–9 years, 10–14 years, and 15–19 years) to improve case definition and reporting.^1^

TB in adolescents has some peculiar characteristics that must be considered when dealing with this age group. Unlike children, whose microbiological diagnosis is difficult to obtain, adolescents more often present clinical forms of TB equal to those found in adults. A higher frequency of positive sputum smear among adolescents, indicative of elevated bacillary load, increases the risk of disease transmission in the community.^13,14^ Besides, adolescents with TB may have comorbidities such as HIV infection, substance abuse, and mental disorders, and usually suffer more from stigma. These factors are associated with a higher risk of TB treatment default.^15,16^

In order to achieve the End TB Strategy objectives, it is necessary to prioritize the most vulnerable individuals, who are often neglected in research agendas and TB control proposals. Adolescents have several peculiarities related to their age group that can compromise their health and adherence to TB treatment;^17,18^ they can thus be characterized as a particularly vulnerable group, in which a person-centered approach becomes even more relevant. Furthermore, adolescents have no strategies for diagnosis and treatment of active or latent TB specific for their age and physical/psychological singularities.

In the present study, we sought to review the works in which the patient-centered approach was used with adolescents, with a particular emphasis on the psychosocial aspects. We hope to offer to health professionals, educators, and public health managers subsidies for a more empathic care, centered on the needs and experiences of adolescents with TB.

## METHOD

The integrative review, chosen for its breadth and scope, has the purpose of grouping and aggregating results of studies on a specific theme, in a systematic and orderly manner, contributing to the deepening of the question investigated and inciting a new understanding on the subject under study.^19–21^

This type of review consists of five stages: formulation of a problem or guiding question, data collection, evaluation of collected data, analysis and interpretation of data, and public presentation of the result.^22^

In the first stage, our guiding question was: is the adolescent affected by TB being assisted according to the principles of person-centered care, with particular attention to the psychosocial aspects involved in this process?

In the second stage, between May and June 2020, we performed data collection, searching in the Virtual Health Library — VHL (LILACS), PubMed (MedLine), and Scopus (Elsevier) databases for manuscripts in Portuguese, Spanish, and English, published from 2000 to 2020. After several refinements in the search, we used the descriptors (DeCS, MeSH) in Portuguese and English, as follows: tuberculosis, adolescent, patient-centered care, and psychology, and to broaden the search we used the keyword ‘psychological support’. This keyword was defined as counseling sessions or peer-group support^8^ and was included to give the necessary balance in document retrieval (Table 1). In order to expand the scope of our research, we performed searches with the same terms on Google Scholar, SciELO, and Web of Science sites; however, we found inconsistent results with low precision level. Consequently, they were not included in this review.

**Table 1:** Search strategies and quantification of the identified records by bibliographic database.

Search strategies	VHL	PubMed	Scopus
Tuberculose AND adolescente AND cuidado centrado no paciente	13	0	0
Tuberculose AND adolescente AND psicologia	370	0	0
Tuberculosis AND adolescent AND psychological support	4	62	49
Tuberculosis AND adolescent AND patient centered care	0	28	47
Tuberculosis AND adolescent AND psychosocial	0	55	74
Tuberculosis AND adolescent AND psychology	0	498	122

VHL: Virtual Health Library.

**Figure 1:**
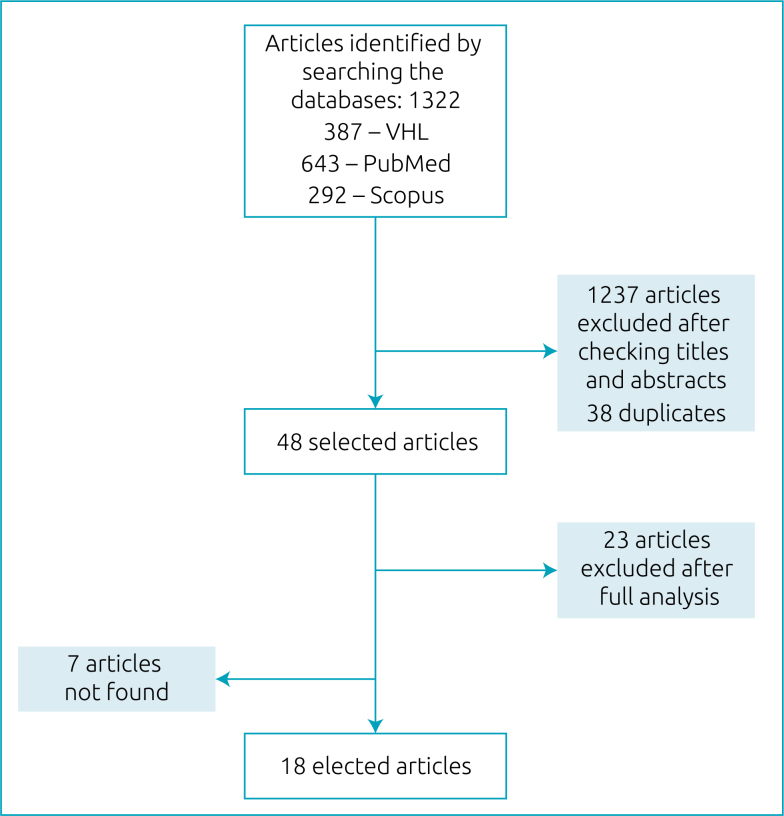
Flowchart of manuscript eligibility.

In the third stage, the following inclusion criteria were used for the selection of the manuscripts retrieved: quantitative and qualitative studies, focus on assistance, educational and programmatic aspects of tuberculosis, which included specific data on adolescents aged 10–19 years. The exclusion criterion was the absence of participants in the age group. Of the total 1322 studies retrieved, 1236 were discarded based on title and abstract reading, and 38 were duplicates. We listed 48 studies for analytical reading; at this point another 23 articles were excluded for being outside the context. Of the remaining 25 documents, seven could not be accessed. Thus, our final selection included 18 articles, of which 12 were quantitative and six were qualitative (Figure 1).

## RESULTS AND DISCUSSION

For the synthesis of evidence, we verified which of the main areas of care for TB patients were addressed in the manuscripts, according to the PCC principles. The main areas identified were the promotion of access to diagnosis, adherence to TB preventive treatment (TPT) and active TB, health education, and psychosocial support to patients and their families.^5,8^

In the fourth stage, we analyzed 18 selected studies. Among the topics mentioned, we found the search for diagnosis; adherence to treatment; assessment of side effects related to TPT and TB treatment; knowledge, attitudes, and practices (KAP) on TB; perception of TB acquisition risks; evaluation of services, and policies in the care of adolescents with TB; educational strategies and experience reports on adolescents with TB.

Many studies were North American (8), published in 2016 (4), had a quantitative methodological approach (12) and four were published in the International Journal of Tuberculosis and Lung Disease. It is also worth noting that we did not find any Brazilian or Portuguese article that fit the selection criteria (Tables 2 and 3).

**Table 2:** Main characteristics of the articles selected between 2000 and 2010.

Authors	Country	Study designs	Objective/intervention	Study population
Morisky et al.,^27^	USA	Quantitative, analytical, randomized, controlled	Impact assessment of four strategies to increase treatment adherence	794 adolescents (11–19 years old) undergoing treatment for latent TB
Hovell et al.,^24^	USA	Quantitative, analytical, randomized, controlled, prospective	Comparison between coaching for adherence and counseling for self-esteem vs. usual medical care	286 adolescents (13–18 years old) undergoing treatment for latent TB
Hovell et al.,^25^	USA	Quantitative, analytical, randomized, controlled, prospective	Assessment of factors associated with treatment adherence related to the study 5	286 adolescents (13–18 years old) undergoing treatment for latent TB
Coly et al,^23^	USA	Quantitative, analytical, randomized, controlled	Analysis of factors associated with treatment completion for latent TB	766 adolescents (11–19 years old) with latent TB
Berg et al.,^29^	USA	Quantitative, analytical, uncontrolled, prospective	Evaluation of side effects and adherence to the treatment of latent TB	96 adolescents (12–19 years old) with latent TB
Blumberg et al.,^30^	USA	Quantitative, analytical, randomized, controlled, prospective	Verification of adherence to the treatment of latent TB through the adolescent's report vs. detection of isoniazid metabolites	286 adolescents (13–18 years old) with latent TB
Cass et al.,^28^	USA	Quantitative, analytical, retrospective	Comparison between usual care vs. treatment behavioral reinforcement intervention to increase adherence	1714 children and adolescents with latent TB, of which 463 were between 10–14 years old
Kominski et al.,^26^	USA	Quantitative, analytical, randomized, controlled	Cost-effectiveness analysis of different strategies for increasing treatment adherence	794 adolescents (11–19 years old) with latent TB
Bond et al.,^38^	Zambia	Qualitative, prospective	Educational intervention based on discussions, drawings, dramatizations, and narratives to promote early case detection	209 adolescents (10–17 years old) without TB

TB: tuberculosis.

**Table 3:** Main characteristics of the articles selected between 2011 and 2020.

Authors	Country	Study designs	Objective/intervention	Study population
Geldenhuys et al.,^36^	South Africa	Quantitative, analytical, transversal	Identification of risk factors for TB among young people with and without TB	292 adolescents (12–18 years old), 62 with active TB, 112 with latent TB and 118 without TB
Isaakidis et al.,^32^	India	Quantitative, descriptive, retrospective	Evaluation of TB treatment of adolescents coinfected with MDR-TB and HIV	11 adolescents (10–19 years old) with MDR-TB
Das et al.,^33^	India	Quantitative, descriptive, retrospective	Psychological support intervention for HIV/TB-MDR coinfected patients with depressive symptoms at the beginning and/or during treatment for MDR-TB	7 people with MDR-TB, being 1 teenager aged 16 years
Alvarez et al.,^37^	Canada	Qualitative, prospective	Educational intervention to promote the performance of adolescents as multipliers of information and awareness about TB	41 young people (12–21 years old) without TB
Schmidt et al.,^39^	South Africa	Qualitative, prospective	Educational intervention with focus groups and role play to raise awareness of TB and increase youth participation in clinical studies	8–15 participants in the focus groups and 7500 reached during the plays, from high school and without TB
Zhang et al.,^35^	China	Qualitative, prospective	Explore, through interviews, the experience of illness of young people with TB	22 high school participants (16–21 years old) with TB
Blok et al.,^40^	Europe	Quantitative, descriptive, cross-sectional	Survey on adolescent TB policies and management among European countries	53 member states of the WHO European Region
Loveday et al.,^34^	South Africa	Qualitative, transversal	Identify, through interviews with caregivers of young people with MDR-TB, the challenges faced during treatment	Caregivers of 26 children and adolescents (aged 2–14 years) with MDR-TB
Amanullah et al.,^41^	N.A.	Qualitative, retrospective	Review of the importance of quality in the care of children and adolescents with TB	Children and adolescents (0–19 years old) with TB

TB: tuberculosis; MDR-TB: multidrug-resistant tuberculosis; HIV: human immunodeficiency virus; WHO: World Health Organization; N.A.: not applicable.

Among the selected studies, a large part (5) addressed the adolescents' adherence to TPT and were from the USA.^23–28^ Among the different strategies used, the most successful and closest to PCC were based on peer counseling, i.e., counseling given by teenagers who completed TPT and were trained to advise and encourage other adolescents on how to use the medication and face the difficulties associated with treatment.

Family collaboration, through parental participation during the different phases of treatment, was also encouraged. In some studies, family participation involved the establishment of agreements, which determined prizes offered when certain stages were overcome, encouraging the adolescent to complete the therapy. Living with both parents, having a positive attitude towards the use of medicines, being bicultural (in the case of Latino teenagers), having higher grades at school, and being young were also factors associated with higher completion of TPT. On the other hand, smoking, alcohol and drug use were associated with lower adherence to TPT.

The relevance of family and culture ties of origin on treatment completion was reinforced in a retrospective article.^28^ The authors compared the adherence to TPT between children and adolescents under the routine care or under the intervention called “Treasure Chest”, a kind of positive reinforcement. Two factors were positively connected with the completion of treatment: participation in the intervention group and the use of the mother tongue (Spanish) in daily life.

Two studies^29,30^ referred to adverse effects secondary to isoniazid use; such events can reduce adherence to TPT, regardless of its severity. Berg et al.^29^ found that the frequency of side effects directly attributable to isoniazid was low and had no significant impact on TPT continuation. However, nonspecific symptoms, such as headache, acne, and dandruff, effects unrelated to medication, negatively affected treatment completion. The authors reinforced the importance of listening to adolescents, allowing health professionals to clarify whether complaints are attributed to medication or not, thus increasing treatment adherence.

On the other hand, Blumberg et al.^30^ compared the reports of medication use with the results of a urine isoniazid metabolite survey in 286 adolescents undergoing TPT, and concluded that the adolescents' information on the correct assumption of TB medication is valid and reliable.

TB treatment requires the combination of four drugs (rifampicin, isoniazid, pyrazinamide, and ethambutol) in the intensive phase (first two months) and two drugs (rifampicin and isoniazid) in the maintenance phase, for a treatment duration of at least six months. However, if the *Mycobacterium tuberculosis* strain is resistant to the main anti-TB drugs, i.e., rifampicin and isoniazid, the so-called multidrug-resistant TB (MDR-TB), the treatment may be as long as 18 to 24 months and requires the combined use of more expensive and toxic drugs, some of them injectable.^31^ Therefore, it is even more necessary to pay attention to the various factors that can lead to suffering and loss of follow-up by the adolescent with MDR-TB. Thus, it was not surprising to find studies in this category concerned with evaluating and promoting biopsychosocial aspects of adolescents' lives.

Isaakidis et al.,^32^ studying 11 adolescents with MDR-TB and HIV coinfection, found that family support during treatment plays a key role in adherence. Most cases had unfavorable outcomes (treatment failure, loss of follow-up, or death) and presented family and social problems in common, such as violence, sexual abuse, low acceptance, and discrimination by the community. Researchers identified that all patients had side effects from the therapies, which highlights the complexity of MDR-TB treatment, particularly when associated with HIV infection. Because of fear of being stigmatized, adolescents did not talk about the disease at school, and as soon as they felt healthier, they tried to return to social life, leaving health care in the background. These results show the relevance of psychological support for adolescents with MDR-TB and HIV coinfection and their families, as well as the role that educational initiatives may have in sensitizing healthcare workers and family members to the need of this support for adolescents.

Das et al.^33^ evaluated the role psychological and, when necessary, psychiatric monitoring may have during treatment of patients with MDR-TB and HIV coinfection. Depression was assessed by the standardized Patient Health Questionnaire-9 (PHQ-9), and the authors observed an improvement in depressive symptoms in six participants, including one adolescent, that had access to psychological assistance. These findings reinforce the need for regular mental health monitoring and psychological support for TB patients, in particular for those with MDR-TB and HIV coinfection, which could also be a strategy to increase adherence to TB treatment.

As previously discussed, the family has a fundamental role in the success of TB treatment in adolescents. The families of these adolescents need to be assisted since diagnosis and treatment determine not only the emotional but also the financial and social impact on them. In addition, since TB is an airborne disease, more than one family member may have TB concurrently, which leads to increased suffering and additional expenses. Loveday et al.^34^ studied children and adolescents under 15 years with MDR-TB/XDR (extensively resistant TB) and their families in a context of high prevalence of HIV coinfection (72%). Families reported that, besides the emotional distress resulting from concerns about the adolescents' health, they suffered financial impact due to expenses for treatment and visits to the hospital. The poorest families had to bear further financial losses and entered into debt. The emotional health of family members was affected, especially that of women, who were the caregivers in 85% of cases. Social protection measures, psychosocial support, and knowledge about the disease are the basis for TB care, not only centered on the patient but on the family as a whole.

Adolescents' perception of TB illness may differ from adults. The impact of TB disease on adolescent's social life and school activities may have greater importance than the physical consequences of the disease itself. In a Chinese study,^35^ 22 high school teenagers with TB reported being more concerned with interrupting their studies than with the fact that they were sick. They showed little knowledge of TB, which led to delayed diagnosis and reduced treatment adherence.

The association between psychosocial and behavioral factors with the risk of adolescents acquiring TB was assessed in South Africa.^36^ A quantitative study was carried out with 292 adolescents (62 with TB, 112 with latent TB, and 118 without TB), using standardized questionnaires to estimate the presence of traumatic events (rape and violence), psychiatric symptoms, social support (family and friends), and substance abuse. There was no evidence of an association between behavioral and psychosocial aspects and TB acquisition. However, adverse experiences in the lives of adolescents with TB were more frequently reported than in the other adolescent groups studied.

TB education can be another approach to PCC with a focus on adolescents, as these young people have a potential role as multipliers of TB knowledge for their peers and communities. However, gaps in knowledge are frequently identified. Some of these gaps can cause stigma and prejudice, as well as delay in diagnosis and low adherence to treatment, generating more suffering for the adolescent patient. Therefore, information about TB, from aspects related to the transmission mechanism to those related to prevention and treatment, constitutes a powerful strategy for the empowerment of adolescents affected by the disease.

Playful activities were strong bets on qualitative studies to invest in adolescents' potential as promoters of health information.^37–39^

An intervention program in Zambia aimed to promote the early detection of TB by having young people as community influencers. A total of 209 adolescents participated in various activities, such as acting, drawing, playing, writing, and discussing TB and HIV.^37^ They showed extensive knowledge of TB and a strong belief in the treatment's effectiveness. However, adolescents still carried stigmatizing beliefs, relating TB to smoking, drinking, mining work, and sex. Despite the fear initially reported by the adolescents, talking to adults about TB was positive and encouraging, which reinforced the role young people can play as promoters of health education in their communities.

Alvarez et al.,^37^ in their study, promoted the participation of the Inuit community in the development and assessment of educational activities on TB. Forty-nine young Inuit participated in these activities, which included the production of videos for later viewing at local community events. Older adolescents showed greater improvements in TB knowledge than younger ones, so they were also more likely to function as independent multipliers of information.

In another study,^39^ adolescents participated in an adaptation of a comic book about TB for a play in schools, and through questionnaires completed by students, the authors observed that adolescents did not know many symptoms, had prejudice against people with TB, and confused aspects of TB with those of HIV infection. However, after the play, the understanding increased and stigma aspects related to TB and HIV diminished. They concluded that playing, more precisely through drama, is a useful educational tool to increase adolescents' awareness and engagement in the fight against TB.

However, knowledge about TB alone is no guarantee that adolescents will seek or have access to diagnosis and treatment. The awareness of health professionals about the importance of TB as a public health problem and the knowledge of preventive and therapeutic measures for the disease offered specifically to adolescents, taking into account their specificities, is an essential aspect that must be addressed together.

Blok et al.^40^ conducted a survey on TB care policies among European adolescents. Of the 28 countries surveyed, only six (21%) had a specific policy for adolescents; of the remaining 22 countries, in nine (32%) adolescents were treated as children and in 13 (46%) as adults. In addition, only 25% of countries perceived adolescents as a group at high risk for TB and only 29% believed that specific guidelines were needed. Among the challenges perceived by these states, health professionals' lack of awareness regarding the specific needs of adolescents and the inadequacy of hospital facilities for this population (aimed at children or adults) were highlighted.

Concerning the quality of care for pediatric TB worldwide, its policies, and academic research, Amanullah et al.^41^ emphasized the lack of specific approaches for non-adult patients and the shortage of academic interventions for young people aged under 17 years. For the researchers, the low quality of care for young people with TB was evident, and there was no focused and friendly approach to children, adolescents and their families. Even with the growing consensus among experts, who recommend the adoption of personalized models for serving children and adolescents with TB, such models are not adopted. Therefore, diagnosis and treatment constantly fall short of the expected result.

Studies addressing the problem of TB among adolescents from the perspective of PCC are still very limited. This finding corroborates what is already observed in clinical and epidemiological studies, where adolescents with TB are still poorly studied as a well-defined age group with their own characteristics. In the selected manuscripts, the division by age group was not defined; we found only one study performed specifically with adolescents from 10–19 years of age.^32^ In addition, some articles were developments of the same main study; in others, the presence of a truly person-centered approach was not easily identifiable.

Among the studies analyzed, those in which the person-centered approach could be more clearly perceived were the ones aimed at promoting adherence to treatment (either TPT or active TB) and qualitative studies that discussed adolescents' KAP on TB and health education. In these studies, it was possible to identify activities in which the adolescents had active participation, their demands were accepted, and they acted as “counselors” for other adolescents or as promoters of education about the disease for the community. These studies demonstrate that education and emotional care strategies enhance adherence, especially when applied by peers.

The positive impact of family support for adolescents affected by TB was another aspect emphasized by the authors. Psychological and financial support to families, especially those with greater social vulnerability, are essential for an effective PCC for these adolescents.

Finally, the importance of psychological support for adolescents with TB, particularly in the presence of comorbidities such as HIV infection, or in the case of MDR/XDR-TB, was an aspect also highlighted in several studies.

However, our review has limitations. The search strategy used, limited to studies that had used or had been indexed with the term PCC, expanding the search only to manuscripts involving the psychosocial aspects of TB in adolescents, may have excluded articles that had not used the term, but followed at least some of the principles of the patient-centered approach. Despite this, the majority of the studies included in this review (11 out of 18) were exclusively about adolescents. Moreover, our search option allowed us to assess how the term is still of limited use in the care of adolescents with TB, and how much the PCC deserves to be expanded, given the positive results achieved in the case of adolescents, as described in the studies presented here.

In summary, this review reinforces the need for studies on adolescents with TB, aged between 10–19 years, and based on the PCC. We believe that investing in this approach will enable adolescents to become more involved in their own treatment, thus mitigating their physical and emotional suffering, and reducing follow-up loss. Within this perspective, adolescents may enhance their social role as TB knowledge multipliers and transformers of the stigmatizing perception of TB in their communities.
